# High-Risk HPV16 E6 Activates the cGMP/PKG Pathway Through Glycosyltransferase ST6GAL1 in Cervical Cancer Cells

**DOI:** 10.3389/fonc.2021.716246

**Published:** 2021-10-20

**Authors:** Jun Wang, Gao Liu, Mei Liu, Qinzhen Cai, Cong Yao, Hao Chen, Neng Song, Chunhui Yuan, Decai Tan, Yuhai Hu, Yun Xiang, Tian Xiang

**Affiliations:** ^1^ Department of Laboratory Medicine, Wuhan Children’s Hospital (Wuhan Maternal and Child Healthcare Hospital), Tongji Medical College, Huazhong University of Science & Technology, Wuhan, China; ^2^ Department of Gastrointestinal Surgery, Central Hospital of Enshi Tujia and Miao Autonomous Prefecture, Enshi Clinical College, Medical School of Hubei Minzu University, Enshi, China; ^3^ Department of Laboratory Medicine, Wuhan Hankou Hospital, Wuhan, China; ^4^ Health Care Department, Wuhan Children’s Hospital (Wuhan Maternal and Child Healthcare Hospital), Tongji Medical College, Huazhong University of Science & Technology, Wuhan, China; ^5^ Department of Pathology, Zhongnan Hospital of Wuhan University, Wuhan, China; ^6^ Department of Laboratory Medicine, Hubei Provincial Hospital of Integrated Chinese & Western Medicine, Wuhan, China; ^7^ Department of Science and Education, Central Hospital of Enshi Tujia and Miao Autonomous Prefecture, Enshi Clinical College, Medical School of Hubei Minzu University, Enshi, China; ^8^ Department of Laboratory Medicine, Central Hospital of Enshi Tujia and Miao Autonomous Prefecture, Enshi Clinical College, Medical School of Hubei Minzu University, Enshi, China

**Keywords:** cervical cancer, E6 protein, YAP1, ST6GAL1, cGMP/PKG

## Abstract

Alterations in glycosylation regulate fundamental molecular and cellular processes of cancer, serving as important biomarkers and therapeutic targets. However, the potential association and regulatory mechanisms of E6 oncoprotein on glycosylation of cervical cancer cells are still unclear. Here, we evaluated the glycomic changes *via* using Lectin microarray and determined the corresponding enzymes associated with endogenous high-risk HPV16 E6 expression in cervical cancer cells. α-2,6 sialic acids and the corresponding glycosyltransferase ST6GAL1 were significantly increased in E6 stable-expressing HPV^−^ cervical cancer C33A cells. Clinical validation further showed that the expression of ST6GAL1 was significantly increased in patients infected with high-risk HPV subtypes and showed a positive association with E6 in cervical scraping samples. Interfering ST6GAL1 expression markedly blocked the oncogenic effects of E6 on colony formulation, proliferation, and metastasis. Importantly, ST6GAL1 overexpression enhanced tumorigenic activities of both E6-positive and E6-negative cells. Mechanistical investigations revealed that E6 depended on activating YAP1 to stimulate ST6GAL1 expression, as verteporfin (inhibitor of YAP1) significantly suppressed the E6-induced ST6GAL1 upregulation. E6/ST6GAL1 triggered the activation of downstream cGMP/PKG signaling pathway and ODQ (inhibitor of GMP production) simultaneously suppressed the oncogenic activities of both E6 and ST6GAL1 in cervical cancer cells. Taken together, these findings indicate that ST6GAL1 is an important mediator for oncogenic E6 protein to activate the downstream cGMP/PKG signaling pathway, which represents a novel molecular mechanism and potential therapeutic targets for cervical cancer.

## Introduction

Cervical cancer remains the fourth most frequently diagnosed cancer and the fourth leading cause of cancer-related deaths among women worldwide (1). The major causative factor that leads to the development of cervical cancer has been identified as human papillomavirus (HPV) persistent infection (2). High-risk HPV16 is the most common carcinogenic HPV subtype and is responsible for more than half of cervical cancers (3–5). Due to the wide coverage of cancer screening and HPV vaccination programs, incidence and mortality rates of cervical cancer have progressively declined in well-developed countries (6), but an increasing incidence and mortality trend is observed in China, especially in young women (7). Furthermore, the vaccine ISA101, one of the most promising therapeutic vaccines that directly targeted the HPV16 E6 and E7 oncoproteins, has been shown to be ineffective for invasive cancer by itself alone (8).

In infected malignant cells, HPV16 genome frequently (>76%) integrated into the host cell DNA (9). The E6 oncoprotein is one major driver of oncogenesis in the normal cervical epithelium and plays important roles for controlling epithelial differentiation, cellular growth, and immune function (10, 11). Mechanistically, E6 forms a complex with host E3 ubiquitin ligases and mediates proteasomal degradation of a number of host targets, such as tumor suppression protein p53 (12), and also increases telomerase activity to prevent apoptosis and promote the immortalization of infected cells (13). Specifically, E6 protein of high-risk HPV16 and HPV18 can further bind and regulate a selection of PSD95/DLG/ZO-1 (PDZ) domain-containing proteins, like DLG1 and SCRIB, to increase oncogenic potential (14). However, HPV16 E6 preferentially binds SCRIB over DLG1 and *vice versa* for HPV18 E6. Furthermore, HPV16 E6 also promotes NHERF1 degradation to enhance the metastasis of cervical cancer cells, whereas NHERF1 is not targeted by HPV18 E6 (15). Recently, disruption of the direct interaction between E6 and host proteins or chimeric antigen receptor T cells (CAR-T cells) targeted to E6 have been shown to be effective for HPV16^+^ cervical cancers (16, 17). Thus, a further detailed clarification of the oncogenic molecular mechanisms of high-risk HPV16 E6 would be beneficial for the development of therapeutic interventions directed against HPV16^+^ malignancies.

Glycosylation alterations frequently occurred in tumor cells and participate in numerous fundamental biological processes, including cellular signaling, proliferation, adhesion, extracellular matrix interactions, proximal and distal communication, inflammation, and immune surveillance (18). For example, with the ability to enhance the biosynthesis of poly-N-acetyllactosamine chains and generation of the backbone components of dimeric sialyl Lewis A, B3GNT3/B3GNT2-mediated glycosylation of both PD-L1 and PD-1 enhances their interaction, and downregulation of these enzymes represents a potential strategy to enhance immune checkpoint therapy (19–21). Sialylation is another important abnormal glycan modification found in the vast majority of cancers, which leads to the hyperactivation of the receptor tyrosine kinases (RTKs), like epidermal growth factor receptor (EGFR), MET, and RON (22, 23). Increased expression of β-galactoside α2,6-sialyltransferase I (ST6GAL1), an enzyme generated α2,6-sialylated lactosamine, is often associated with invasive phenotype and poor prognosis in several cancers, including colon, stomach, and ovarian cancers (24). Moreover, ST6GAL1 endows resistance of cancer cells to gefitinib (EGFR inhibitor) and trastuzumab (anti-ErbB2 antibody) and also predicts the sensitivity of lenvatinib treatment to patients with hepatocellular carcinoma (25). Thus, targeting glycosylation alterations holds potent promise to augment the available strategies in both therapy and biomarker field of cancers.

Recently, increased sialylation, GlcNAcylation, and reduced fucosylation have been found to be upregulated in carcinomas of the cervix (26–28) and patients with metastatic cervical cancer (29). However, the glycosylation alterations of cervical cancer in response to high-risk HPV16 E6 stimulation, and the potential regulatory mechanisms remain largely unknown. In the present study, we confirmed that E6 increases level of α2-6 sialic acids in cervical cancer HPV^−^ C33A cells *via* using Lectin microarray, and ST6GAL1 is an important mediator for E6 to perform oncogenic activities. In details, E6 depends on activating YAP1 to stimulate ST6GAL1 expression and subsequently results in the activation of downstream cGMP/PKG pathway. Importantly, knockdown ST6GAL1 or blocking cGMP/PKG pathway with specific inhibitor ODQ suppressed the oncogenic activities of both E6-positive and E6-negative cervical cancer cells. Therefore, our findings provide novel insights into the molecular mechanisms involved in HPV16 E6-induced oncogenesis and suggest that targeting ST6GAL1 or cGMP–PKG signaling might be potential therapeutic strategies for cervical cancer.

## Materials and Methods

### Cell Culture

The human embryonic kidney (HEK293T) cells, C33A (HPV^−^) cells, were purchased from the cell bank of the Chinese Academy of Sciences (Shanghai, China) as we previously described (30). HEK293T cells and C33A and C33A-E6 cells were grown in Dulbecco’s modified Eagle’s medium (DMEM)–low glucose (Gibco, USA) containing 10% fetal bovine serum (FBS) (Gibco, USA), 100 U/ml penicillin, and 100 µg/ml streptomycin (Beyotime, Shanghai, China) under a humidified atmosphere containing with 5% CO_2_ at 37°C.

### Collection of Cervical Specimens

Samples of all patients were collected with a clinically approved disposable cervical abscission cell sampler (158-0001, Kang Jian Medical, Jiangsu, China). The instructions for use of these cervical sampling brush were as follows: expose the cervix, wipe off the secretions with a cotton swab, carefully insert the brush head into the endocervical canal until the outer bristles touch the outer cervical neck, rotate clockwise five to eight times, and finally insert the brush head into the preservation solution bottle. Genomic DNA was first extracted from the collected samples using a DNA extraction kit (Chaozhou Hybribio Biochemical Co., Ltd., Chaozhou, China) according to the manufacturer’s instructions. According to the clinical detection results of HPV typing as we presented in our previous study (31), 10 samples of five groups were collected respectively and stored at −80°C, and then, ST6GAL1 and E6 gene tests were performed. The five groups including (1) single HPV16-positive specimens, (2) mixed HPV16-positive specimens, (3) other high-risk HPV-positive specimens, (4) low-risk HPV-positive specimens, and (5) HPV-negative specimens. The overview of the clinical characteristics of patients are shown in **Supplementary Table S1**. The study was approved by the Institutional Review Board of the Wuhan Children’s Hospital (Wuhan Maternal and Child Healthcare Hospital) (2020R079-E01).

### Lentivirus Production and Transduction

HPV16 E6 stable-expressing C33A cells were constructed according to our previous study (32). In brief, HPV16 E6 (GenBank accession no. FJ415228.1) was cloned into the lentiviral backbone pLVX-mCMV-ZsGreen-IRES-Puro. Then, the plasmids, pCAG-HIVgp (RDB04394, Riken, Japan) and pCMV-VSV-G-RSV-Rev (RDB04393, Riken, Japan), were simultaneously transfected into HEK293T using Lipofectamine 2000 (Invitrogen, Shanghai, China). Lentiviral particles in the supernatant were harvested at 72 h after transfection. C33A cells were then infected with pLVX-E6 or control pLVX lentivirus [multiplicity of infection (MOI) = 50] in the presence of 8 μg/ml polybrene. The cells were treated with puromycin (5 μg/ml) for 2 weeks to select the stably transfected cells.

### Lectin Microarray

A lectin microarray was produced using 95 lectins (Raybiotech Lectin Array 95 kit, Cat. No. GA-Lectin-95, RayBiotech, Inc.) with different binding preferences covering *N-* and *O*-linked glycans and analyzed as previously described (33, 34). Whole-cell lysates labeled with fluorescent dye Cy3 (GE Healthcare, UK) were applied to the lectin microarrays, which were then scanned with a Genepix 4000B confocal scanner (Axon Instruments, CA, USA). The acquired images were analyzed at 532 nm for Cy3 detection by Genepix 3.0 software (Axon Instruments, Inc.). Raw values of fluorescent intensities less than the average background were removed. The median of the valid data for each lectin was globally normalized to the sum of the medians of all valid data for 95 lectins. The relative change in protein glycosylation in response to HPV16 E6 stimulation was evaluated by comparison of the data from E6 stable-expressing versus control C33A cell lysates.

### Transient Transfection

Short-hairpin RNA targeting human ST6GAL1 (pSilencer 1.0-U6-shST6GAL1) and negative control shRNA (pSilencer 1.0-U6-shNC) were designed and chemically synthesized by the TSINGKE Biological Technology (Wuhan, China). According to the targeting sequences, oligonucleotides coding for each short-hairpin RNA (shRNA) were designed and listed as follows: shRNA ST6GAL1, forward 5′-GGGAGTTACTATGATTCCTTTTTCAA GCTTAAAGGAATCATAGTAACTCCCTTTTT-3′; reverse, 5′-AATTAAAAAGGG AGTTACTATGATTCCTTTAAGCTTGAAAAAGGAATCATAGTAACTCCCGGCC -3′); shRNA-negative control (NC) (forward, 5′-TTCTCCGAACGTGTCACGTTCAA GCTTCGTGACACGTTCGGAGAATTTTT-3′; reverse, 5′-AATTAAAAATTCTCC GAACGTGTCACGAAGCTTGAACGTGACACGTTCGGAGAAGGCC-3′). Messenger RNA (mRNA) of ST6GAL1 (GenBank accession no. NM_173216.2) was synthesized and inserted into the pcDNA3.1 vector (Invitrogen, Carlsbad, CA, USA); empty vector transfection was transfected as the control. C33A or C33A-E6 cells were grown to a density about 70–80% in 6-cm dishes and then transfected with indicated plasmids by Lipofectamine 2000 Transfection Kit (Invitrogen, Shanghai, China) following the manufacturers’ instruction. Forty-eight hours posttransfection, the selective overexpression or silencing of ST6GAL1 was detected by Western blot.

### mRNA Sequencing

Total RNA was extracted from C33A and C33A-E6 cells with Trizol reagent (Invitrogen, CA, USA). Then, the RNA was quantified using a NanoDrop ND-2000 (Thermo Scientific), and its integrity was assessed using the Agilent Bioanalyzer 2100 (Agilent Technologies, USA). Next, the mRNA molecules were purified from the total RNA using oligo (dT) magnetic beads and fragmented into small pieces and then subjected to transcriptome analysis (OEbiotech, Shanghai, China). The E6-dysregulated mRNAs were identified by the combination of the absolute value of the |log2 [fold change of reads per kilo base per million mapped reads (RPKM)]| ≥ 1 and *p* < 0.05. ST6GAL1-associated mRNAs were analyzed using R2: Genomics Analysis and Visualization Platform (http://r2.amc.nl) as we previously described (32); the cutoff correlation *p*-value was <0.01. Cluster Profiler R package was used for Kyoto Encyclopedia of Genes and Genomes (KEGG) pathway enrichment analysis. MEV software was used for hierarchical cluster analysis of mRNA expression according to the protocol provided by OEbiotech.

### Quantitative Real-Time PCR

Total RNA extracted from indicated cervical cancer cells with Trizol reagent (Invitrogen, CA, USA). Subsequently, complementary DNA was generated from 1 μg total RNA using Prime Script RT Master Mix (Takara Bio Inc., Kusatsu, Japan). Real-Time PCR (qRT-PCR) was conducted using 2× SYBR green PCR master mix (TaKaRa, Dalian, China) according to the manufacturer’s protocol to detect mRNA levels of indicated genes. The ABI 7500 (Applied Biosystems, CA, USA) was used to perform the amplification reaction. Each experiment was performed in triplicate. Gene expression values were normalized relative to expression of the housekeeping gene glyceraldehyde 3-phosphate dehydrogenase (GAPDH) by the 2^−ΔΔCt^ method. The gene-specific primer sequences are listed in **Table 1**.

**Table 1:** Primer sequences for genes.

Gene	Forward primer (5′-3′)	Reversed primer (5′-3′)
*HPV16 E6*	ATGCACCAAAAAGGAACTGCAATGT	TTACAGCTGGGTTTCTCTACGTGTT
*B3GALNT1*	CTCCTGAGTTTCTTTGTGATGTGG	CATTACGTACTTGGCATTGGGG
*B4GALNT1*	GGAACCTGGCCGTGTCTCAAGTAAC	CCAGGAAGAAGTTAACCACGCCGTC
*ST6GAL1*	CTACCATTCGCCTGATGAACTCT	TCTGGGGCTTGAGGATGTAAAAG
*ST6GAL2*	CTGAATGGGAGGGTTATCTGCC	ACCTCAGGACTGCGTCATGATC
*MAN2A1*	TTAAGCCGCCAGTTCACCG	ACATTGAGAGCTGGCCCTGAG
*B4GALT1*	TGAGTTTAACATGCCTGTGGACCTG	AATGAGGTCCACGTCACTAAACAC
*FUT1*	ATTAGGTGACAAGCGGGCAGAGGC	TTCCGGTGCCAGGGCTTAGAG
*FUT2*	GTCACCGATGCTGGAAGGGTTT	GTCCCAGTGCCTTTGATGTTGAG
*B3GNT3*	TATGTGTCTGGAGCTTGAGG	AAGGATGTGTAGGAGTTCGC
*B3GNT2*	CTGCTCCCGGACAAGATATGA	GTACTGCCGGTTCAGCTTCT
*GAPDH*	GGGAGCCAAAAGGGTCAT	GAGTCCTTCCACGATACCAA

### Western Blot

Cells were lysed on ice with radioimmunoprecipitation assay (RIPA) buffer (Beyotime, China) containing a protease inhibitor cocktail. The concentration of protein was detected by a BCA Protein Assay Kit (Beyotime). Protein samples (30 µg) were separated by 8–12% (v/v) sodium dodecyl sulfate polyacrylamide (SDS-PAGE) gel electrophoresis and transferred onto a polyvinylidene fluoride (PVDF) membrane (Millipore, MA, USA) for subsequent experiments. The membranes were blocked by 5% defatted milk for 2 h at room temperature and then incubated with primary antibodies against HPV16E6 (sc-460; 1:1,000; 16 kDa; Santa Cruz), ST6GAL1 (14355-1-AP; 1:1,000; 47 kDa; Proteintech), B3GNT2 (sc-134231; 1:1,000; 46 kDa; Santa Cruz), FUT2 (sc-100742, 1:1,000; 39 kDa; Santa Cruz), B4GALT1 (sc-515551, 1:1,000; 50 kDa; Santa Cruz), Bcl-2 (ab196495; 1:1,000; 26 kDa; Abcam), Bax (50599-2-lg; 1:10,000; 21 kDa; Proteintech), p53 (ab26; 1:1,000; 53 kDa; Abcam), YAP1 (ab56701; 1:1,000; 49 kDa; Abcam), p-YAP1^S397^ (#13619; 1:1,500; 75 kDa; CST), VASP (13472-1-AP; 1:1,000; 46 kDa; Proteintech), p-VASP^Ser239^ (ab194747; 1:1,000; 40 kDa; Abcam), Smad2 (ab119907; 1:1,000; 52 kDa; Abcam), p-Smad2^S467^ (ab53100; 1:1,000; 58 kDa; Abcam), E-cadherin (13-1700; 1:1,000; 110 kDa; Invitrogen), sGC (ab189176, 1:1,000; 71 kDa; Abcam), PKG1 (#3248, 1:1,000; 48 kDa; CST), PKG2 (55138-1-AP; 1:1,000; 87 kDa; Proteintech), PDE5A (ab28761, 1:1,000; 95 kDa; Abcam), and GAPDH (sc-47724; 1:1,000; 37 kDa; Santa Cruz) at 4°C overnight. Next, the membranes were incubated with horseradish peroxidase (HRP)-conjugated secondary antibodies and visualized by chemiluminescence (Pierce ECL Western Blotting Substrate).

### Cell Counting Kit-8 Assay

Cells (1.5 × 10^3^) were seeded into 96-well plates and incubated with 5% CO_2_ at 37°C, after incubation for 24, 48, 72, and 96 h, 10 μl Cell Counting Kit-8 (CCK-8) reagent (Yeasen, Shanghai, China) was added into each well and incubated for another 4 h. Cell viability was assessed by detecting the absorbance of the dye solution at 450 nm in a microplate reader (Bio-Rad, CA, USA). All samples were prepared in triplicate and normalized to a blank control.

### Colony Formation Assay

Cells (1 × 10^3^) were plated in six-well plates for 24 h and then received indicated transfection or drug treatment. Cells were then cultured in fresh medium for another week. Colonies were fixed with methanol and stained with crystal violet (0.05%, w/v) for 30 min. Photographs were acquired, and colonies containing more than 50 cells were counted. All the experiments were performed in triplicate.

### Cell Apoptosis Assay

For cell apoptosis analysis, cells were incubated with Annexin V-FITC and propidium iodide using an Annexin V-FITC Apoptosis Detection Kit (Biolegend) according to the manufacturer’s protocol at 4°C in the dark for 30 min. The fluorescence of the stained cells was examined by flow cytometry (FACSCanto II, Becton-Dickinson, USA) and analyzed by FlowJo 10.5.4 software.

### Wound-Healing Assay

A wound-healing assay was used to evaluate the migration of C33A and C33A-E6 cells. Briefly, cells (1 × 10^5^) were seeded in 24-well plates for growth to 80–90% confluence. Then, the monolayers were wounded by scratching the surface as uniformly as possible with a pipette tip. The remaining cells were washed thrice in phosphate-buffered saline (PBS) to remove cellular debris and incubated at 37°C with serum-free medium. Migrating cells at the wound front were photographed under an Olympus IX73 microscope after 24 h. The area of the wound was measured with ImageJ software (NIH, USA).

### Transwell Assay

Transwell assay was used to investigate cells invasion in C33A and C33A-E6 cells as we described previously (30, 32). Briefly, 24-well Transwell plates (pore size, 8 µm; Corning, Inc.) with 100 µl Matrigel (BD Biosciences) in upper chamber were used for cell invasion assays. Cells (5 × 10^4^) were resuspended in serum-free medium and placed into the upper chamber. Complete medium in the lower chamber was considered as a chemical attractant. After incubating for 24 h, the invaded cells attached to the lower surface were fixed with 4% formaldehyde for 15 min and then stained with 0.05% crystal violet for 30 min. The invasion cells were photographed under an Olympus IX73 microscope. Experiments were performed in three independent times and six random fields were scanned in each time.

### Statistical Analysis

The data were presented as mean ± standard deviation of at least three independent experiments and analyzed by GraphPad Prism 8.0 Software (GraphPad Software Inc., La Jolla, CA, USA). Unpaired *t*-test or one-way ANOVA followed by Newman–Keuls *post-hoc* test was performed for data analysis. A *p* < 0.05 was considered as statistically significant (*p < 0.05, **p < 0.01, ***p < 0.001).

## Results

### The High-Risk HPV16 E6 Protein Results in Altered Glycosylation in Cervical Cancer Cells

**Figure 1:**
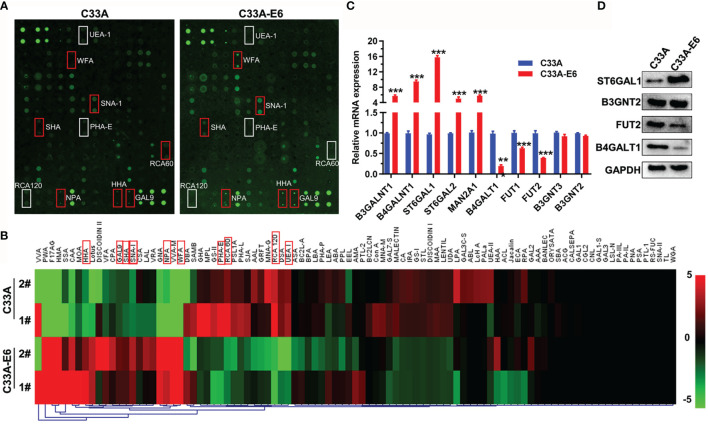
The high-risk HPV16 E6 protein results in altered glycosylation in cervical cancer cells. **(A)** C33A cells were infected with pLVX-E6 or empty control pLVX lentivirus (MOI = 50) to select the stably transfected cells with the presence of puromycin (5 μg/ml). Then, glycosylation changes in whole-cell lysates of C33A and C33A-E6 cells (E6 stable expressing) were detected by lectin microarray (included 95 lectins). The slide with two replicates of the lectin array is shown. The lectin microarrays with high fluorescence intensities in C33A-E6 cells are marked with red frames, and low fluorescence intensities in C33A-E6 cells are marked with white frames. **(B)** Fluorescence intensities of samples from C33A and C33A-E6 cells were scanned and analyzed. Heat map and hierarchical clustering analysis of the 95 lectins with two biological replicates were presented. The significant different lectins were marked with red frames. **(C)** qRT-PCR was performed to detect the mRNA expression of lectin-corresponding glycotransferases of C33A and C33A-E6 cells. Data were presented as mean ± SD from the three independent replicates. **(D)** Protein expression of several glycotransferases were confirmed by Western blot. **p < 0.01, ***p < 0.001.

To examine the relationship between HPV16 E6 and altered glycosylation in cervical cancer, we constructed HPV16 E6-stable expressing HPV^−^ C33A cervical cancer cells (C33A-E6) and detected the glycan repertoire of cellular glycoproteins *via* using 95-lectins microarray. In general, C33A-E6 cells showed higher levels of α-2,6 sialic acids (bound by lectin SNA-I), N-acetylgalactosamine (WFA, SHA), α-1,3- and α-1,6-linked mannose (HHA, NPL), β-galactosidase (GAL9), and lower levels of lactose/galactose (RCA120, RCA60, PHA-E), and α-1,2 fucose structures (UEA-I) than the corresponding control C33A cells (**Figures 1A, B**; **Table 2**). To gain insight into the biosynthetic underpinnings of these changes, we then focused on glycogenes (glycosyltransferases) known to be involved in the synthesis of glycan epitopes bound by these lectins. Consistent with our observed glycan changes, C33A-E6 cells displayed higher expression levels of N-acetylgalactosamine branching enzymes (B3GALNT1 and B4GALNT1), sialyltransferases (ST6GAL1 and ST6GAL2), and α-1,3- and α-1,6-linked mannose extension enzymes (MAN2A1) than control C33A cells, and lower levels of lactose/galactose branching enzyme B4GALT1 and α-1,2 mannosidases (FUT1 and FUT2) (**Figures 1C, D**). In addition, poly-N-acetyllactosamine (DSA, WGA) and its corresponding enzymes (B3GNT2 and B3GNT3) showed no changes (**Figures 1A–D**). These results indicate that E6 results in altered glycosylation in cervical cancer cells. Given the strong evidence for a relationship between the glycosylation and cancer progression (18), we hypothesized that altered expression of these glycogenes and their corresponding changes in glycan epitopes may be involved in the oncogenic activities of HPV16 E6.

**Table 2:** Differences in glycan patterns between C33A and C33A-E6 cells by lectin microarray analysis.

Lectin	Abbreviation	Carbohydrate specificity	Glycosyltransferase	Fold change (C33A-E6 vs. C33A)	*p*
*Sambucus nigra* I	SNA-I	α-2,6 sialic acid	ST6GAL1, ST6GAL2	3.13	0.0099
*Wisteria floribunda*	WFA	GalNAc	B3GALNT1, B4GALNT1	2.62	0.022
*Salivia horminum lectin*	SHA	GalNAc	B3GALNT1, B4GALNT1	1.11	0.0106
*Hippeastrum hybrid*	HHA	α-1,3-Linked mannose	MAN2A1	1.95	0.0299
*Narcissus pseudonarcissus*	NPA	α-1,6-Linked mannose	MAN2A1	2.19	0.0179
*Ulex europaeus* I	UEA-I	α-1,2-Fucose structures	FUT1, FUT2	0.58	0.0364
*Ricinus communis agglutinin* I	RCA 120	Galactose, lactose	B4GALT1	0.17	0.005
*Ricinus communis agglutinin* II	RCA 60	Galactose, lactose	B4GALT1	0.46	0.0191
*Phaseolus vulgaris erythroagglutinin*	PHA-E	galactose	B4GALT1	0.64	0.0238

### ST6GAL1 Is a Pivotal Oncogenic Mediator of the High-Risk HPV16 E6

**Figure 2:**
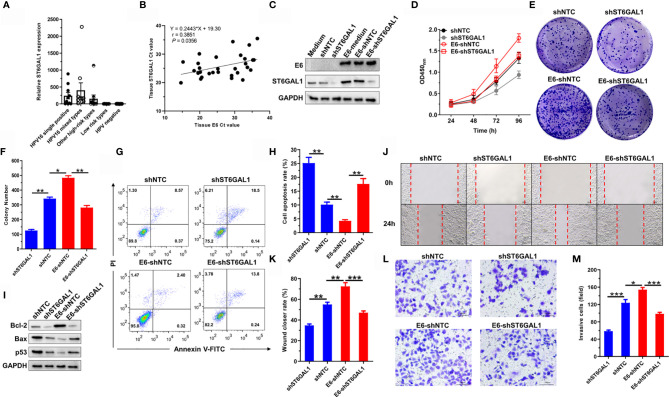
ST6GAL1 is a pivotal oncogenic mediator of the high-risk HPV 16 E6. **(A)** Analysis of ST6GAL1 expression in cervical scraping samples of five patient groups (n = 10 in each group). HPV-negative samples were used as control group, and ST6GAL1 expression values were normalized relative to expression of the housekeeping gene GAPDH by the 2^−ΔΔCt^ method. **(B)** The association of ST6GAL1 to E6 in E6-detected samples was analyzed by Spearman analysis (n = 30). **(C)** C33A and C33A-E6 (E6 stable expressing) cells were transiently transfected with short hairpin RNA targeting ST6GAL1 (shST6GAL1) or a non-targeting control (shNTC). Then, protein expression of E6 and ST6GAL1 were detected by Western blot. **(D)** Proliferation of indicated cells were detected by CCK-8 assay. **(E)** The clones of indicated cells were visualized by crystal violet staining. **(F)** Statistical analysis of colony numbers in C33A and C33A-E6 cells. **(G, H)** Apoptosis of indicated cells were detected by flow cytometry, and then, apoptotic rates were statistically analyzed. **(I)** Western blot was used to detect the expression of apoptosis-related proteins. **(J, K)** Migrative abilities of indicated cells were detected by wound-healing assay and statistically analyzed. **(L, M)** Invasive abilities of indicated cells were detected by Transwell assay and statistically analyzed. Data were presented as mean ± SD from the three independent replicates. *p < 0.05; **p < 0.01; ***p < 0.001.

Knockdown of ST6GAL1 suppresses subcutaneous tumor growth and increases cisplatin sensitivity of cervical cancer cells (35); we therefore presumed that ST6GAL1 may be a candidate oncogenic mediator of HPV16 E6 protein. To validate this speculation, we first detected ST6GAL1 expression in cervical scraping samples of patients with different subtypes of HPV infection. The expression of ST6GAL1 was significantly higher in patients infected with high-risk HPV subtypes, including HPV16^+^, HPV18^+^, or others (**Figure 2A**). Moreover, Ct value of ST6GAL1 showed a positive association with E6 in E6-detected cervical scraping samples (**Figure 2B**). Then, ST6GAL1 was silenced in both C33A and C33A-E6 cells *via* transient transfecting with short-hairpin RNA targeting ST6GAL1 (shST6GAL1) or a non-targeting control (shNTC) (**Figure 2C**). CCK-8 and clonogenic assays revealed that ST6GAL1 knockdown markedly attenuated the oncogenic effects of E6 on cellular proliferation (**Figure 2D**) and colony formulation (**Figures 2E, F**). Comparing to shNTC-transfected C33A-E6 cells, the apoptotic rate (**Figures 2G, H**) and expression of proapoptotic proteins, Bax and p53, were also significantly increased in cells transfected with shST6GAL1, and antiapoptotic factor Bcl-2 was significantly decreased (**Figure 2I**). Furthermore, ST6GAL1 knockdown significantly attenuated the metastatic potential of C33A-E6, including migration (**Figures 2J, K**) and invasion (**Figures 2L, M**) in wound-scratching and Transwell assays. Therefore, these data demonstrate that ST6GAL1 is a pivotal mediator for E6 to perform oncogenic activities in cervical cancer cells.

### ST6GAL1 Promotes Oncogenic Activities in Both E6-Positive and E6-Negative Cervical Cancer Cells

**Figure 3:**
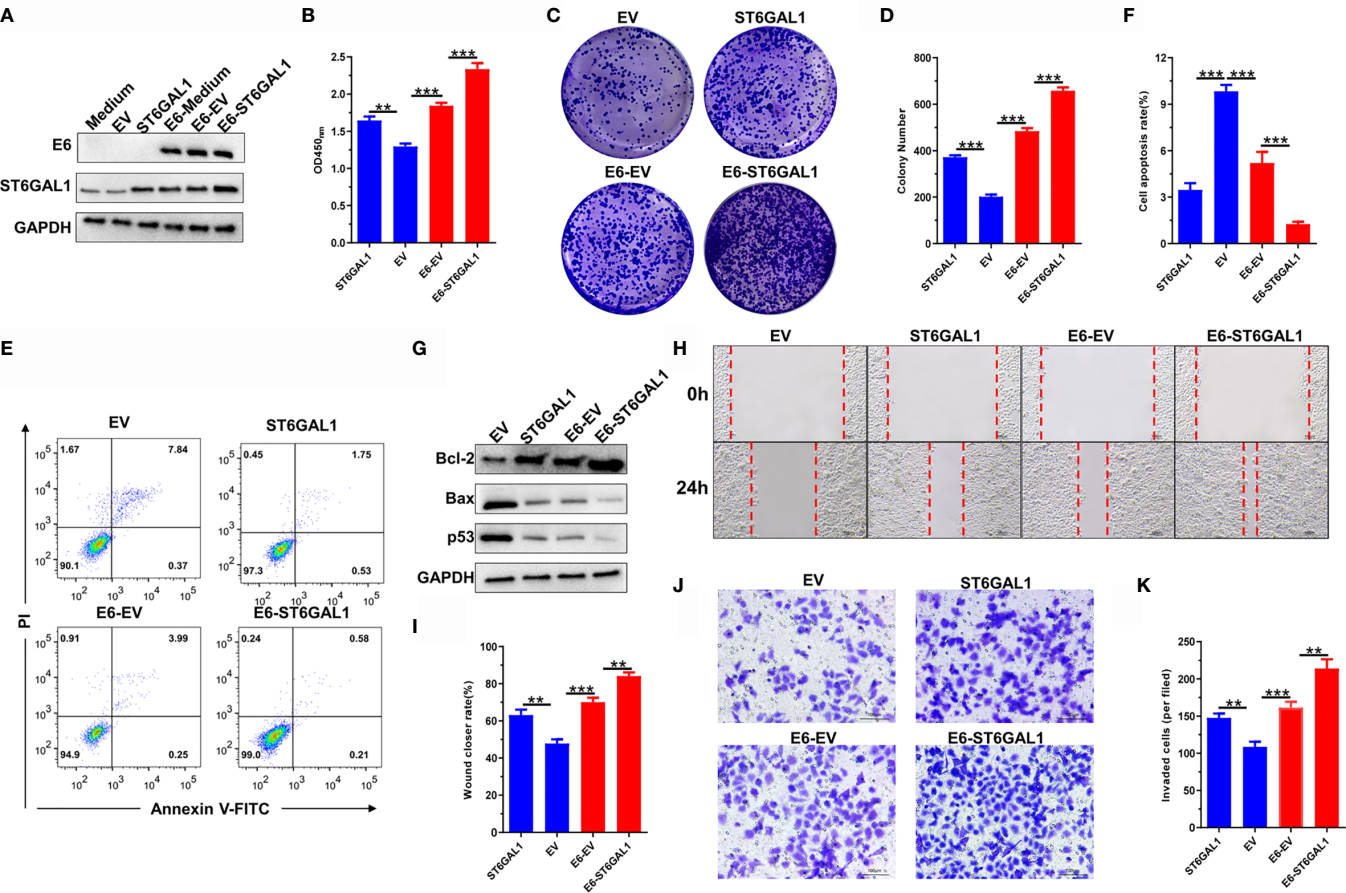
ST6GAL1 promotes oncogenic activities in both E6-positive and E6-negative cervical cancer cells. **(A)** C33A and C33A-E6 (E6 stable expressing) cells were transiently transfected with pcDNA3.1-ST6GAL1 overexpressing plasmid (ST6GAL1) or empty vector (EV). Then, protein expression of E6 and ST6GAL1 were detected by Western blot. **(B)** Proliferation of indicated cells were detected by CCK-8 assay. **(C)** The clones of indicated cells were visualized by crystal violet staining. **(D)** Statistical analysis of colony numbers in C33A and C33A-E6 cells. **(E, F)** Apoptosis of indicated cells were detected by flow cytometry, and then, apoptotic rates were statistically analyzed. **(G)** Western blot was used to detect the expression of apoptosis-related proteins. **(H, I)** Migrative abilities of indicated cells were detected by wound-healing assay and statistically analyzed. **(J, K)** Invasive abilities of indicated cells were detected by Transwell assay and statistically analyzed. Data were presented as mean ± SD from the three independent replicates. **p < 0.01; ***p < 0.001.

Knockdown of ST6GAL1 suppresses the oncogenic activities of HPV18^+^ Hela cells (35) and also HPV^−^ C33A cells (**Figure 2**). To further confirm the oncogenic potential of ST6GAL1 with or without the status of HPV infection, we then transiently overexpressed ST6GAL1 in both C33A and C33A-E6 cells (**Figure 3A**). Cellular proliferation (**Figure 3B**) and colony formulation (**Figures 3C, D**) of both C33A and C33A-E6 cells were significantly increased in response to ST6GAL1 overexpression. In addition, overexpression of ST6GAL1 also significantly decreased the apoptotic rates (**Figures 3E, F**) and the expression of proapoptotic proteins, Bax and p53 (**Figure 3G**) in both C33A and C33A-E6 cells. Antiapoptotic protein Bcl-2 was significantly increased in response to ST6GAL1 overexpression (**Figure 3G**). With regard to the metastatic potential, ST6GAL1 overexpression significantly increased migrating (**Figures 3H, I**) and invading (**Figures 3J, K** **)** abilities of both C33A and C33A-E6 cells as revealed in wound-scratching and Transwell assays. Taken together, these data demonstrate a crucial oncogenic role of ST6GAL1 in both E6-positive and E6-negative cervical cancer cells.

### HPV16 E6 Promotes ST6GAL1 Expression by Activating YAP1

**Figure 4:**
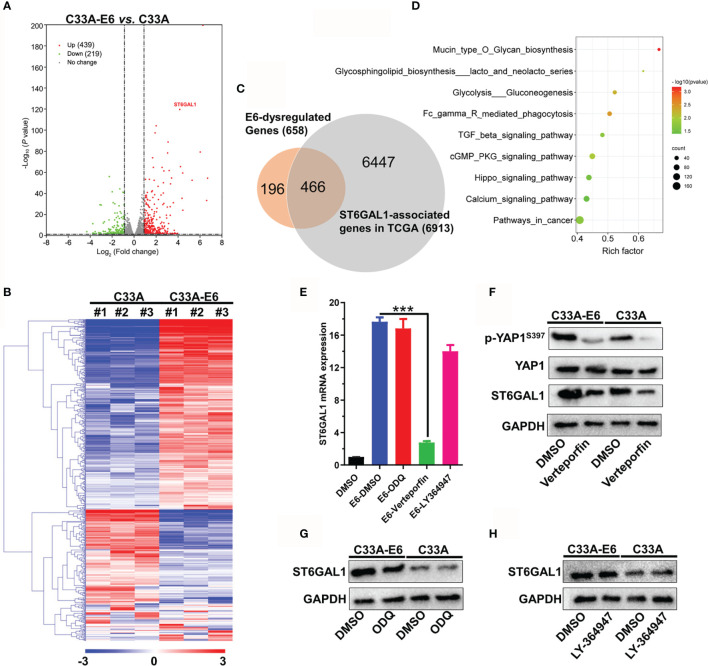
HPV16 E6 promotes ST6GAL1 expression by activating Hippo signaling pathway. **(A)** Dysregulated mRNAs between C33A and C33A-E6 (E6 stable expressing) cells were detected by mRNA sequencing. **(B)** Heat map and hierarchical clustering analysis of 658 dysregulated mRNAs with three independent biological replicates were presented. **(C)** Venn diagram of 658 E6-dysregulated mRNAs and 6,913 ST6GAL1-asscociated mRNAs in TCGA dataset (N = 305). ST6GAL1-associated mRNAs were identified using R2: Genomics Analysis and Visualization Platform (http://r2.amc.nl). **(D)** KEGG enrichment analysis of 466 mRNAs that simultaneously associated with E6 and ST6GAL1. **(E)** C33A and C33A-E6 cells were treated with verteporfin (5 μM, Hippo signaling inhibitor), ODQ (10 μM, cGMP/PKG inhibitor), and LY-364947 (5 μM, TGF-β inhibitor) for 24 h, and then, qRT-PCR was used to detect ST6GAL1 mRNA expression. DMSO was used as solvent control. ST6GAL1 protein expression of C33A and C33A-E6 cells in response to **(F)** verteporfin, **(G)** ODQ, and **(H)** LY-364947 treatment was detected by Western blot. Data were presented as mean ± SD from the three independent replicates. ***p < 0.001.

To identify the potent regulatory mechanism of HPV16 E6 on ST6GAL1, mRNA sequencing was performed on both C33A and C33A-E6 cells, and the results showed that E6-stable expression resulted in dysregulation of 658 mRNAs, including 439 upregulated and 219 downregulated mRNAs (**Figures 4A, B**). Importantly, 466 of 658 mRNAs were significantly associated with ST6GAL1 expression in TCGA cervical cancer dataset (**Figure 4C**). KEGG enrichment analysis revealed that these 466 mRNAs were mainly involved in glycan biosynthesis, pathways in cancer, and TGF-β, cGMP/PKG, and Hippo signaling pathways (**Figure 4D**). Next, selective enzyme inhibitors were used to examine which signaling pathway mediated the upregulation of ST6GAL1 in C33A-E6 cells. The mRNA expression of ST6GAL1 was significantly suppressed with the presence of verteporfin, an inhibitor of YAP1 in the Hippo signaling, whereas cGMP/PKG inhibitor ODQ and TGF-β inhibitor LY-364947 showed no obvious effects (**Figure 4E**). E6 stable expression in C33A cells significantly suppressed Ser397-phosphorylated YAP (degradation status) and enhanced total YAP expression (**Figure 4F**). Consistent with mRNA results, only the addition of verteporfin significantly downregulated ST6GAL1 protein expression in both C33A and C33A-E6 cells (**Figure 4F**) but not ODQ (**Figure 4G**) and LY-364947 (**Figure 4H**). Thus, these findings provide compelling evidence that activation of YAP1 is critical to ST6GAL1 expression and is a major mediator of E6-induced ST6GAL1 upregulation.

### ST6GAL1 Activates cGMP/PKG Signal Axis to Play Oncogenic Activities

**Figure 5:**
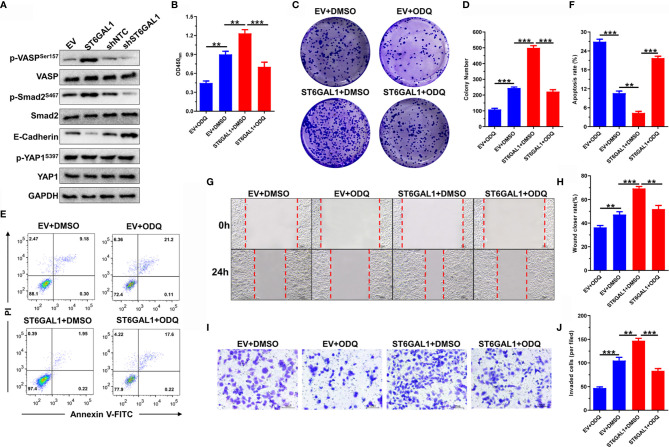
ST6GAL1 activates cGMP/PKG signal axis to play oncogenic activities. **(A)** C33A cells were transiently transfected with pcDNA3.1-ST6GAL1 overexpressing plasmid (ST6GAL1), empty vector (EV), short hairpin RNA targeting ST6GAL1 (shST6GAL1), or a non-targeting control (shNTC). Forty-eight hours later, expression of classic proteins involved in TGF-β, cGMP/PKG, and Hippo signaling pathways were detected by Western blot. **(B)** Proliferation of indicated cells were detected by CCK-8 assay. **(C)** The clones of indicated cells were visualized by crystal violet staining. **(D)** Statistical analysis of colony numbers in C33A cells received indicated treatment. **(E, F)** Apoptosis of indicated cells were detected by flow cytometry, and then, apoptotic rates were statistically analyzed. **(G, H)** Migrative abilities of indicated cells were detected by wound-healing assay and statistically analyzed. **(I, J)** Invasive abilities of indicated cells were detected by Transwell assay and statistically analyzed. Data were presented as mean ± SD from the three independent replicates. **p < 0.01; ***p < 0.001.

We next examined the effects of ST6GAL1 on TGF-β, cGMP/PKG, and Hippo signaling pathways. The phosphorylation of VASP (a major effector indicates cGMP/PKG signaling activation) and Smad was significantly increased by ST6GAL1 overexpression and *vice versa* for ST6GAL1 knockdown (**Figure 5A**). The expression of E-cadherin was significantly reduced, and YAP1 expression showed no response to ST6GAL1 overexpression (**Figure 5A**). These results indicated that ST6GAL1 activates both cGMP/PKG and TGF-β signaling. Activation of cGMP/PKG promotes chemoresistance and maintenance of cancer stem cells in cervical, breast, ovarian, and gastric cancers (32, 36). Thus, we further investigated the therapeutic potential of cGMP/PKG inhibitor ODQ on ST6GAL1 overexpression. The ST6GAL1-enhanced cellular proliferation (**Figure 5B**) and colony formulation (**Figures 5C, D**) of C33A cells were significantly suppressed by ODQ treatment. In addition, ODQ also significantly promoted apoptosis (**Figures 5E, F**) and blocked the migration (**Figures 5G, H**) and invasion (**Figures 5I, J**) of ST6GAL1-overexpressing cells. Taken together, these results demonstrate that ST6GAL1 depends on activating cGMP/PKG signaling axis to play oncogenic activities in C33A cervical cancer cells.

### HPV16 E6 Activates cGMP/PKG Signaling Pathway Through ST6GAL1

**Figure 6:**
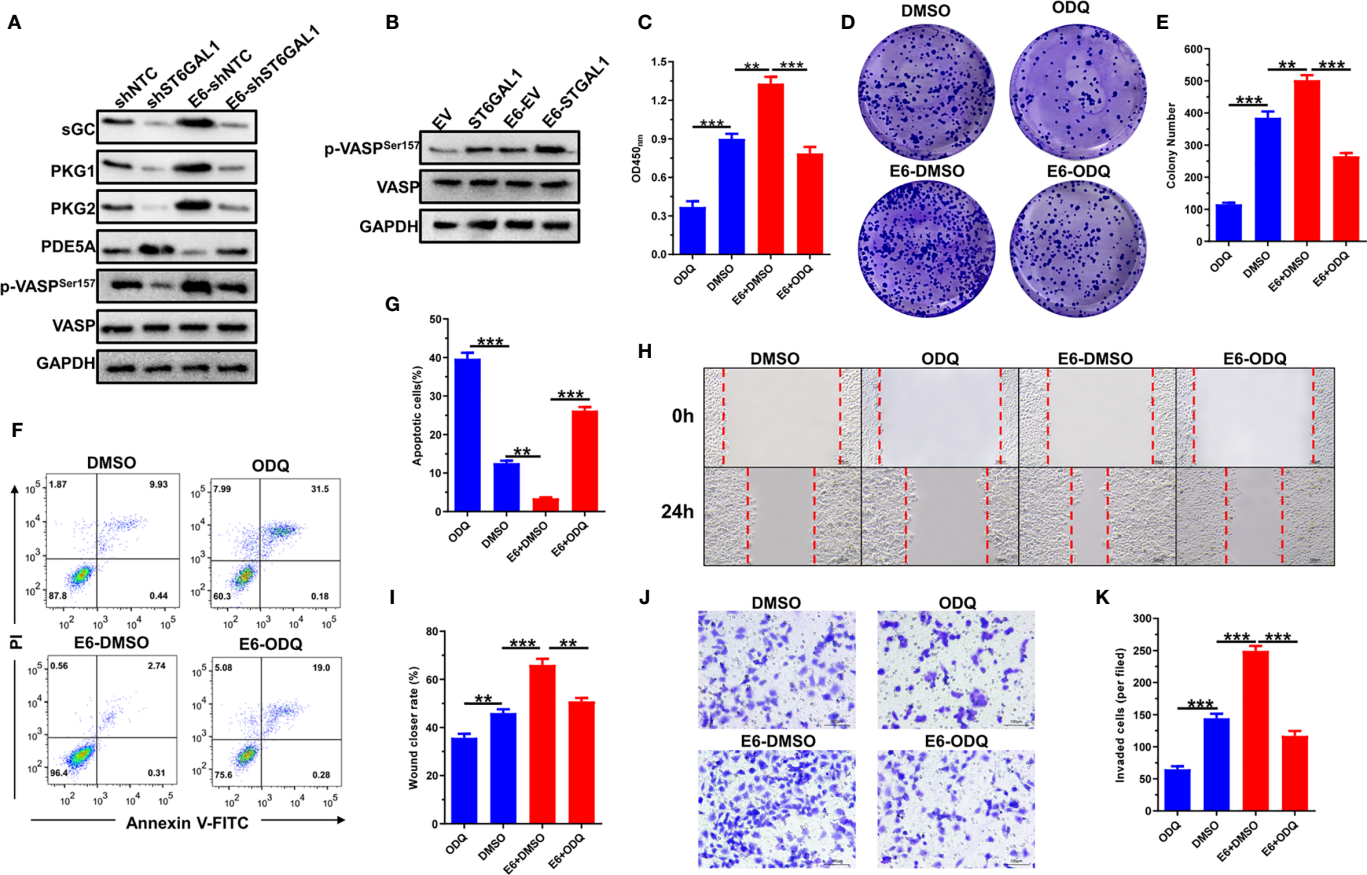
HPV16 E6 activates cGMP/PKG signaling pathway through ST6GAL1. **(A)** C33A and C33A-E6 (E6 stable expressing) cells were transiently transfected with short hairpin RNA targeting ST6GAL1 (shST6GAL1) or a non-targeting control (shNTC). Forty-eight hours later, the expression of cGMP/PKG signaling pathway-related proteins were detected by Western blot. **(B)** C33A and C33A-E6 cells were transiently transfected with pcDNA3.1-ST6GAL1 overexpressing plasmid (ST6GAL1) or empty vector (EV). Forty-eight hours later, phosphorylated VASP expression in indicated cells were detected by Western blot. **(C)** Proliferation of indicated cells were detected by CCK-8 assay. **(D)** The clones of indicated cells were visualized by crystal violet staining. **(E)** Statistical analysis of colony numbers in indicated cells. **(F, G)** Apoptosis of indicated cells were detected by flow cytometry, and then, apoptotic rates were statistically analyzed. **(H, I)** Migrative abilities of indicated cells were detected by wound-healing assay and statistically analyzed. **(J, K)** Invasive abilities of indicated cells were detected by Transwell assay and statistically analyzed. Data were presented as mean ± SD from the three independent replicates. **p < 0.01; ***p < 0.001.

The activation of cGMP/PKG signaling plays oncogenic roles in both HPV16^+^ Siha and HPV18^+^ Hela cells (37). Thus, we further evaluated whether HPV16 E6 activates cGMP/PKG signaling and the role of ST6GAL1 in this process. As expected, the molecules indicate that cGMP/PKG signaling activation, including sGC, PKG1, PKG2, PDE5A, and p-VASP, was significantly upregulated by E6-stable expression in HPV^−^ C33A cells (**Figure 6A**). ST6GAL1 knockdown markedly attenuated E6-induced activation of cGMP/PKG signaling, and its overexpression further elevated p-VASP levels in C33A-E6 cells (**Figure 6B**). These results indicate that HPV16 E6 activates cGMP/PKG signaling in cervical cancer cells, and ST6GAL1 is critical for this process. The effects of ODQ on E6-mediated oncogenic activities were next evaluated, and the results revealed that ODQ significantly suppressed cellular proliferation (**Figure 6C**) and colony formulation (**Figures 6D, E**) in both C33A and C33A-E6 cells. The apoptotic rates were also increased in response to ODQ treatment (**Figures 6F, G**). Furthermore, the migration (**Figures 6H, I**) and invasion (**Figures 6J, K**) of both C33A and C33A-E6 cells were significantly decreased with the presence of ODQ. Taken together, these data demonstrate a requirement for ST6GAL1-cGMP/PKG signaling axis in oncogenic properties of E6 and supports its inhibition as a viable therapeutic strategy against cervical cancer.

## Discussion

HPV16 E6 oncoprotein plays multifunctional roles on post-translational modifications (PTMs), including ubiquitination, acetylation, and phosphorylation, and thereby contributes to the viral life cycle and to the induction of malignancy (38–40). Serving as an important kind of PTMs, glycosylation can act as a key regulatory mechanism to control cancer progression and highlight its application in the clinical setting as appealing targets for personalized medicine (41). However, the glycosylation alterations that associated with HPV infection and their role in controlling the progression of cervical cancer remain largely unknown. In the present study, our results confirmed that HPV16 E6 oncoprotein induces significant glycosylation alterations in HPV^−^ C33A cells *via* using 95-lectins microarray, including increased expression of α-2,6 sialic acids (corresponding glycotransferases, ST6GAL1, ST6GAL2), α-1,3- and α-1,6-linked mannose (MAN2A1), and N-acetylgalactosamine (B3GALNT1, B4GALNT1), and decreased levels of lactose/galactose (B4GALT1) and α-1,2 fucose structures (FUT1, FUT2).

Remarkably, numerous studies have confirmed that these glycosylation alterations induced by E6 stable expression play important roles in modulating cancer signaling, tumor progression, metastasis, antitumor immunity, and responses of current cancer-targeted therapies (18). For example, FUT1 and FUT2 promote growth, adhesion, migration, and cancer stem cell (CSC) properties of breast cancer (42). Increased expression of B4GALNT1 promotes metastasis of lung adenocarcinoma and melanoma (43, 44). B4GALT1 upregulates glycosylation of CDK11^p110^ and therefore confers chemoresistance of pancreatic ductal adenocarcinomas (45). Although poly-N-acetyllactosamine and its corresponding glycotransferases (B3GNT2 and B3GNT3) that facilitate PD-L1/PD-1 stabilization and interaction (19–21) showed no changes in response to exogenous E6 expression, the enhanced MAN2A1 expression has been reported to result in the dysfunction of T cells in tumor microenvironment, and its inhibition enhances the immune response to anti-PD-L1 in human tumors (46). More importantly, HPV16 or HPV18 E6 enhances c-MYC stability *via* promoting its *O*-GlcNAcylation on Thr58 (28). Knockdown of O-GlcNAc transferase (OGT) or ST6GAL1 suppresses tumorigenesis and metastasis of HPV18^+^ cervical cancer Hela cells (28, 35). Hence, alteration of glycosylation is apparently a crucial step for the E6 to initiate and promote cervical cancer progression and urgently needs to be clarified in future.

ST6GAL1, responsible for the terminal α2,6-sialylation of *N*-glycans, can be upregulated by HPV16 E5 oncoprotein, and its inhibition increases cisplatin sensitivity of HPV18^+^ cervical cancer cells (35, 47). In the present study, we found that ST6GAL1 expression in cervical scraping samples is significantly increased in patients infected with high-risk HPV subtypes (HPV16, HPV18, HPV52, etc.) compared to low-risk subtypes or HPV-negative samples. Because all the samples with various groups of patients (HPV positive and/or negative) were collected from Asian origin, this could represent a limitation. For further studies, possibly multicenter (e.g., American Indian or Alaska Native, Indian, Black or African American, and White ethnicity) and with a larger number of patients (especially in developing countries where cervical cancer is still widespread) will be enrolled. Furthermore, ST6GAL1 positively associates with E6 expression in cervical samples and serves as an important oncogenic mediator in both HPV16 E6-positive and E6-negative C33A cervical cancer cells. Due to having a type I PDZ binding motif (PBM) at the C-terminus, high-risk HPV E6 proteins interacts with many PDZ proteins, like PTPN3, LRCC1, and DLG1, and thus promotes the nuclear localization of transcriptional coactivator YAP1, the major effector of the Hippo pathway (48). Through mRNA sequencing and associated molecular analysis, we found that YAP1 is a pivotal mediator for E6 to induce ST6GAL1 expression. Taken together, we thus speculated that ST6GAL1 may have an important role in cervical cancer, especially in high-risk HPV-induced carcinogenesis.

Hippo/YAP signaling pathway plays a critical role in the progression of cervical cancer and can further interact with the ErbB2 and EGFR RTKs to form a positive feedback autocrine/paracrine loop (49). ST6GAL1 endows resistance of cancer cells to gefitinib (EGFR inhibitor) and trastuzumab (anti-ErbB2 antibody) (25) and also confers tumor cell resistance against hypoxia *via* enhancing hypoxia-inducible factor-1α (HIF-1α) expression (50). Recently, molecular analysis has confirmed that ST6GAL1 decreases the sensitization of cancer cells to trastuzumab-induced cytotoxicity through directly enhancing α2,6-sialylation of ErbB2, which results in the increased activation of both ErbB2 and EGFR RTKs (51). Thus, ST6GAL1 may serve as a linker that bridges Hippo/YAP signaling and RTKs activation. With the fact that hyperactivation of genes involved in Hippo/YAP signaling–RTKs-positive feedback loop occurring in most advanced/recurrent cervical cancer patients (>70%) (9, 49, 52) and directly targeting the HPV-16 E6 has been shown to be ineffective for invasive cancer by itself alone (8), target inhibition of ST6GAL1 may be a promising combinational therapeutic strategy for RTK inhibitors or E6-directed therapies in cervical cancer.

In the present study, since we did not perform in-depth mass spectrometry-based glycomic and glycoproteomic analysis, the exact target proteins of ST6GAL1 cannot be confirmed. However, we found that ST6GAL1 activates cGMP/PKG and TGF-β signaling according to the data obtained in mRNA sequencing and association analysis. Aberrant activation of cGMP/PKG signaling promotes chemoresistance, maintenance of cancer stem cells, and cell survival in various human cancers, including lung, cervical, breast, ovarian, and gastric cancers (32, 37). Moreover, we found that blocking cGMP/PKG signaling with ODQ eliminates the oncogenic activities of both E6 and ST6GAL1 in cervical cancer cells. The puzzled question is that activation of cGMP/PKG signaling induces MST/LATS kinases, resulting in the phosphorylation and cytosolic degradation of YAP1 in prostate cancer cells (53), while HPV16 E6 has been showed to have little or no effect on the LATS1/2 (49). Thus, E6 may play a sophisticated role in the progression of cervical cancer, and the detailed molecular targets of ST6GAL1 need to be further identified for unveiling its contribution in E6-induced carcinogenesis.

## Conclusion

In this paper, we demonstrated that high-risk HPV16 E6 oncoprotein induced significant glycosylation alterations in cervical cancer cells. In-depth analyses further revealed that α2,6-sialyltransferase, ST6GAL1, was a novel E6-induced oncogenic protein and performed oncogenic activities in both E6-positive and E6-negative cervical cancer cells. E6 depended on YAP1 to stimulate ST6GAL1 expression and subsequently resulted in the activation of downstream cGMP/PKG pathway. Most importantly, knockdown ST6GAL1 or target inhibition of cGMP/PKG pathway suppressed colony formulation and metastasis of cervical cancer cells. Therefore, ST6GAL1 or cGMP/PKG signaling might be novel targets for the development of drugs against cervical cancer.

## Data Availability Statement

The datasets presented in this study can be found in online repositories. The names of the repository/repositories and accession number(s) can be found in https://portal.gdc.cancer.gov/.

## Ethics Statement

The studies involving human participants were reviewed and approved by Institutional Review Board of the Wuhan Children’s Hospital (Wuhan Maternal and Child Healthcare Hospital). Written informed consent for participation was not required for this study in accordance with the national legislation and the institutional requirements.

## Author Contributions

DT, YH, YX, and TX: study conception and design. JW, GL, ML, QC, CYao, and HC: data acquisition. CYuan and NS: analysis and data interpretation. JW and GL: drafting of the manuscript. All authors contributed to the article and approved the submitted version.

## Funding

This study was supported by the National Natural Science Foundation of China (81760540 and 82060539), Natural Science Foundation of Wuhan Municipal Health Commission (grant no. WX18Q27), Natural Science Foundation of Hubei Municipal Health Commission (grant no. WJ2021M016), The Top Medical Young Talents of Hubei Province (2016), and The Open Research Fund Program of the State Key Laboratory of Virology of China (grant no. 2015KF010).

## Conflict of Interest

The authors declare that the research was conducted in the absence of any commercial or financial relationships that could be construed as a potential conflict of interest.

## Publisher’s Note

All claims expressed in this article are solely those of the authors and do not necessarily represent those of their affiliated organizations, or those of the publisher, the editors and the reviewers. Any product that may be evaluated in this article, or claim that may be made by its manufacturer, is not guaranteed or endorsed by the publisher.

## References

[1] Sung H Ferlay J Siegel RL Laversanne M Soerjomataram I Jemal A. Global Cancer Statistics 2020: GLOBOCAN Estimates of Incidence and Mortality Worldwide for 36 Cancers in 185 Countries. CA Cancer J Clin (2021) 71(3):209–49. doi: 10.3322/caac.21660 33538338

[2] Olusola P Banerjee HN Philley JV Dasgupta S. Human Papilloma Virus-Associated Cervical Cancer and Health Disparities. Cells (2019) 8(6):622. doi: 10.3390/cells8060622 PMC6628030 31234354

[3] National Institutes of Health. Virus Genome Variants. In PapillomaVirus Episteme. Bethesda, MA, USA: NIH. (2019). Available at: https://pave.niaid.nih.gov/#.

[4] Bhatt KH Neller MA Srihari S Crooks P Lekieffre L Aftab BT. Profiling HPV-16-Specific T Cell Responses Reveals Broad Antigen Reactivities in Oropharyngeal Cancer Patients. J Exp Med (2020) 217(10):e20200389. doi: 10.1084/jem.20200389 32716518 PMC7537390

[5] Long W Yang Z Li X Chen M Liu J Zhang Y. HPV-16, HPV-58, and HPV-33 Are the Most Carcinogenic HPV Genotypes in Southwestern China and Their Viral Loads Are Associated With Severity of Premalignant Lesions in the Cervix. Virol J (2018) 15(1):94. doi: 10.1186/s12985-018-1003-x 29801461 PMC5970451

[6] Buskwofie A David-West G Clare CA. A Review of Cervical Cancer: Incidence and Disparities. J Natl Med Assoc (2020) 112(2):229–32. doi: 10.1016/j.jnma.2020.03.002 32278478

[7] Chen W Zheng R Baade PD Zhang S Zeng H Bray F. Cancer Statistics in China, 2015. CA Cancer J Clin (2016) 66(2):115–32. doi: 10.3322/caac.21338 26808342

[8] Massarelli E William W Johnson F Kies M Ferrarotto R Guo M. Combining Immune Checkpoint Blockade and Tumor-Specific Vaccine for Patients With Incurable Human Papillomavirus 16-Related Cancer: A Phase 2 Clinical Trial. JAMA Oncol (2019) 5(1):67–73. doi: 10.1001/jamaoncol.2018.4051 30267032 PMC6439768

[9] Burk RD Chen Z Saller C Tarvin K Carvalho AL Scapulatempo-Neto C. Integrated Genomic and Molecular Characterization of Cervical Cancer. Nature (2017) 543(7645):378–84. doi: 10.1038/nature21386 PMC5354998 28112728

[10] Kuguyo O Tsikai N Thomford NE Magwali T Madziyire MG Nhachi CFB. Genetic Susceptibility for Cervical Cancer in African Populations: What Are the Host Genetic Drivers? OMICS (2018) 22(7):468–83. doi: 10.1089/omi.2018.0075 30004844

[11] Tewari KSS M W Long HJ. Improved Survival With Bevacizumab in Advanced Cervical Cancer. N Engl J Med (2017) 377(7):702. doi: 10.1056/NEJMx170002 28745937

[12] Li S Hong X Wei Z Xie M Li W Liu G. Ubiquitination of the HPV Oncoprotein E6 Is Critical for E6/E6AP-Mediated P53 Degradation. Front Microbiol (2019) 10:2483. doi: 10.3389/fmicb.2019.02483 31749782 PMC6842930

[13] Katzenellenbogen R. Telomerase Induction in HPV Infection and Oncogenesis. Viruses (2017) 9(7):180. doi: 10.3390/v9070180 PMC5537672 28698524

[14] Thomas M Myers MP Massimi P Guarnaccia C Banks L. Analysis of Multiple HPV E6 PDZ Interactions Defines Type-Specific PDZ Fingerprints That Predict Oncogenic Potential. PloS Pathog (2016) 12(8):e1005766. doi: 10.1371/journal.ppat.1005766 27483446 PMC4970744

[15] Wang Q Song R Zhao C Liu H Yang Y Gu S. HPV16 E6 Promotes Cervical Cancer Cell Migration and Invasion by Downregulation of NHERF1. Int J Cancer (2019) 144(7):1619–32. doi: 10.1002/ijc.31876 30230542

[16] Draper LM Kwong ML Gros A Stevanović S Tran E Kerkar S. Targeting of HPV-16+ Epithelial Cancer Cells by TCR Gene Engineered T Cells Directed Against E6. Clin Cancer Res (2015) 21(19):4431–9. doi: 10.1158/1078-0432.ccr-14-3341 PMC4603283 26429982

[17] Celegato M Messa L Goracci L Mercorelli B Bertagnin C Spyrakis F. A Novel Small-Molecule Inhibitor of the Human Papillomavirus E6-P53 Interaction That Reactivates P53 Function and Blocks Cancer Cells Growth. Cancer Lett (2020) 470:115–25. doi: 10.1016/j.canlet.2019.10.046 31693922

[18] Mereiter S Balmaña M Campos D Gomes J Reis CA. Glycosylation in the Era of Cancer-Targeted Therapy: Where Are We Heading? Cancer Cell (2019) 36(1):6–16. doi: 10.1016/j.ccell.2019.06.006 31287993

[19] Lee HH Wang YN Xia W Chen CH Rau KM Ye L. Removal of N-Linked Glycosylation Enhances PD-L1 Detection and Predicts Anti-PD-1/PD-L1 Therapeutic Efficacy. Cancer Cell (2019) 36(2):168–78.e4. doi: 10.1016/j.ccell.2019.06.008 31327656 PMC6793936

[20] Li CW Lim SO Chung EM Kim YS Park AH Yao J. Eradication of Triple-Negative Breast Cancer Cells by Targeting Glycosylated PD-L1. Cancer Cell (2018) 33(2):187–201.e10. doi: 10.1016/j.ccell.2018.01.009 29438695 PMC5824730

[21] Sun L Li CW Chung EM Yang R Kim YS Park AH. Targeting Glycosylated PD-1 Induces Potent Antitumor Immunity. Cancer Res (2020) 80(11):2298–310. doi: 10.1158/0008-5472.can-19-3133 PMC7272274 32156778

[22] Liu Y-C Yen H-Y Chen C-Y Chen C-H Cheng P-F Juan Y-H. Sialylation and Fucosylation of Epidermal Growth Factor Receptor Suppress Its Dimerization and Activation in Lung Cancer Cells. Proc Natl Acad Sci USA (2011) 108(28):11332–7. doi: 10.1073/pnas.1107385108 PMC3136320 21709263

[23] Mereiter S Magalhães A Adamczyk B Jin C Almeida A Drici L. Glycomic Analysis of Gastric Carcinoma Cells Discloses Glycans as Modulators of RON Receptor Tyrosine Kinase Activation in Cancer. Biochim Biophys Acta (2016) 1860(8):1795–808. doi: 10.1016/j.bbagen.2015.12.016 26721331

[24] Wichert B Milde-Langosch K Galatenko V Schmalfeldt B Oliveira-Ferrer L. Prognostic Role of the Sialyltransferase ST6GAL1 in Ovarian Cancer. Glycobiology (2018) 28(11):898–903. doi: 10.1093/glycob/cwy065 30016515

[25] Myojin Y Kodama T Maesaka K Motooka D Sato Y Tanaka S. ST6GAL1 Is a Novel Serum Biomarker for Lenvatinib-Susceptible FGF19-Driven Hepatocellular Carcinoma. Clin Cancer Res (2021) 27(4):1150–61. doi: 10.1158/1078-0432.ccr-20-3382 33288659

[26] Jin Y Kim SC Kim HJ Ju W Kim YH Kim HJ. Increased Sialylation and Reduced Fucosylation of Exfoliated Cervical Cells Are Potential Markers of Carcinogenesis in the Cervix. Clin Chem Lab Med (2016) 54(11):1811–9. doi: 10.1515/cclm-2015-1014 27092648

[27] Rivera-Juarez Mde L Rosas-Murrieta NH Mendieta-Carmona V Hernandez-Pacheco RE Zamora-Ginez I Rodea-Avila C. Promoter Polymorphisms of ST3GAL4 and ST6GAL1 Genes and Associations With Risk of Premalignant and Malignant Lesions of the Cervix. Asian Pac J Cancer Prev (2014) 15(3):1181–6. doi: 10.7314/apjcp.2014.15.3.1181 24606438

[28] Zeng Q Zhao RX Chen J Li Y Li XD Liu XL. O-Linked GlcNAcylation Elevated by HPV E6 Mediates Viral Oncogenesis. Proc Natl Acad Sci USA (2016) 113(33):9333–8. doi: 10.1073/pnas.1606801113 PMC4995993 27482104

[29] Wang PH Lee WL Lee YR Juang CM Chen YJ Chao HT. Enhanced Expression of Alpha 2,6-Sialyltransferase ST6Gal I in Cervical Squamous Cell Carcinoma. Gynecol Oncol (2003) 89(3):395–401. doi: 10.1016/s0090-8258(03)00127-6 12798701

[30] Wang J Xiang F Liu X Ma X Cai X Yang Y. HPV E7 Affects the Function of Cervical Cancer Cells via the TAL1/lnc−EBIC/KLHDC7B Axis. Oncol Rep (2021) 45(5):51. doi: 10.3892/or.2021.8002 33760214

[31] Xiang F Guan Q Liu X Xiao H Xia Q Liu X. Distribution Characteristics of Different Human Papillomavirus Genotypes in Women in Wuhan, China. J Clin Lab Anal (2018) 32(8):e22581. doi: 10.1002/jcla.22581 29862560 PMC6220820

[32] Xiang T Yuan C Guo X Wang H Cai Q Xiang Y. The Novel ZEB1-Upregulated Protein PRTG Induced by Helicobacter Pylori Infection Promotes Gastric Carcinogenesis Through the cGMP/PKG Signaling Pathway. Cell Death Dis (2021) 12(2):150. doi: 10.1038/s41419-021-03440-1 33542225 PMC7862680

[33] Qin Y Zhong Y Zhu M Dang L Yu H Chen Z. Age- and Sex-Associated Differences in the Glycopatterns of Human Salivary Glycoproteins and Their Roles Against Influenza A Virus. J Proteome Res (2013) 12(6):2742–54. doi: 10.1021/pr400096w 23590532

[34] Tang XL Yuan CH Ding Q Zhou Y Pan Q Zhang XL. Selection and Identification of Specific Glycoproteins and Glycan Biomarkers of Macrophages Involved in Mycobacterium Tuberculosis Infection. Tuberculosis (Edinb) (2017) 104:95–106. doi: 10.1016/j.tube.2017.03.010 28454656

[35] Zhang X Pan C Zhou L Cai Z Zhao S Yu D. Knockdown of ST6Gal-I Increases Cisplatin Sensitivity in Cervical Cancer Cells. BMC Cancer (2016) 16(1):949. doi: 10.1186/s12885-016-2981-y 27986075 PMC5162090

[36] Klutzny S Anurin A Nicke B Regan JL Lange M Schulze L. PDE5 Inhibition Eliminates Cancer Stem Cells via Induction of PKA Signaling. Cell Death Dis (2018) 9(2):192. doi: 10.1038/s41419-017-0202-5 29416006 PMC5833477

[37] Gong L Lei Y Tan X Dong Y Luo Z Zhang D. Propranolol Selectively Inhibits Cervical Cancer Cell Growth by Suppressing the cGMP/PKG Pathway. BioMed Pharmacother (2019) 111:1243–8. doi: 10.1016/j.biopha.2019.01.027 30841438

[38] Chen X Loo JX Shi X Xiong W Guo Y Ke H. E6 Protein Expressed by High-Risk HPV Activates Super-Enhancers of the EGFR and C-MET Oncogenes by Destabilizing the Histone Demethylase KDM5C. Cancer Res (2018) 78(6):1418–30. doi: 10.1158/0008-5472.can-17-2118 29339538

[39] Basukala O Sarabia-Vega V Banks L. Human Papillomavirus Oncoproteins and Post-Translational Modifications: Generating Multifunctional Hubs for Overriding Cellular Homeostasis. Biol Chem (2020) 401(5):585–99. doi: 10.1515/hsz-2019-0408 31913845

[40] Niebler M Qian X Höfler D Kogosov V Kaewprag J Kaufmann AM. Post-Translational Control of IL-1β via the Human Papillomavirus Type 16 E6 Oncoprotein: A Novel Mechanism of Innate Immune Escape Mediated by the E3-Ubiquitin Ligase E6-AP and P53. PloS Pathog (2013) 9(8):e1003536. doi: 10.1371/journal.ppat.1003536 23935506 PMC3731255

[41] Pinho SS Reis CA. Glycosylation in Cancer: Mechanisms and Clinical Implications. Nat Rev Cancer (2015) 15(9):540–55. doi: 10.1038/nrc3982 26289314

[42] Lai TY Chen IJ Lin RJ Liao GS Yeo HL Ho CL. Fucosyltransferase 1 and 2 Play Pivotal Roles in Breast Cancer Cells. Cell Death Discov (2019) 5:74. doi: 10.1038/s41420-019-0145-y 30854233 PMC6403244

[43] Yoshida H Koodie L Jacobsen K Hanzawa K Miyamoto Y Yamamoto M. B4GALNT1 Induces Angiogenesis, Anchorage Independence Growth and Motility, and Promotes Tumorigenesis in Melanoma by Induction of Ganglioside GM2/GD2. Sci Rep (2020) 10(1):1199. doi: 10.1038/s41598-019-57130-2 31988291 PMC6985110

[44] Jiang T Wu H Lin M Yin J Tan L Ruan Y. B4GALNT1 Promotes Progression and Metastasis in Lung Adenocarcinoma Through JNK/c-Jun/Slug Pathway. Carcinogenesis (2021) 42(4):621–30. doi: 10.1093/carcin/bgaa141 33367717

[45] Chen Y Su L Huang C Wu S Qiu X Zhao X. Galactosyltransferase B4GALT1 Confers Chemoresistance in Pancreatic Ductal Adenocarcinomas by Upregulating N-Linked Glycosylation of CDK11(P110). Cancer Lett (2021) 500:228–43. doi: 10.1016/j.canlet.2020.12.006 33309857

[46] Shi S Gu S Han T Zhang W Huang L Li Z. Inhibition of MAN2A1 Enhances the Immune Response to Anti-PD-L1 in Human Tumors. Clin Cancer Res (2020) 26(22):5990–6002. doi: 10.1158/1078-0432.ccr-20-0778 32723834 PMC8500537

[47] Cisneros-Ramírez D Martínez-Laguna Y Martínez-Morales P Aguilar-Lemarroy A Jave-Suárez LF Santos-López G. Glycogene Expression Profiles From a HaCaT Cell Line Stably Transfected With HPV16 E5 Oncogene. Mol Med Rep (2020) 22(6):5444–53. doi: 10.3892/mmr.2020.11630 PMC7647045 33174037

[48] Webb Strickland S Brimer N Lyons C Vande Pol SB. Human Papillomavirus E6 Interaction With Cellular PDZ Domain Proteins Modulates YAP Nuclear Localization. Virology (2018) 516:127–38. doi: 10.1016/j.virol.2018.01.003 PMC5826896 29346075

[49] He C Mao D Hua G Lv X Chen X Angeletti PC. The Hippo/YAP Pathway Interacts With EGFR Signaling and HPV Oncoproteins to Regulate Cervical Cancer Progression. EMBO Mol Med (2015) 7(11):1426–49. doi: 10.15252/emmm.201404976 PMC4644376 26417066

[50] Jones RB Dorsett KA Hjelmeland AB Bellis SL. The ST6Gal-I Sialyltransferase Protects Tumor Cells Against Hypoxia by Enhancing HIF-1α Signaling. J Biol Chem (2018) 293(15):5659–67. doi: 10.1074/jbc.RA117.001194 PMC5900773 29475939

[51] Duarte HO Rodrigues JG Gomes C Hensbergen PJ Ederveen ALH de Ru AH. ST6Gal1 Targets the Ectodomain of ErbB2 in a Site-Specific Manner and Regulates Gastric Cancer Cell Sensitivity to Trastuzumab. Oncogene (2021) 40(21):3719–33. doi: 10.1038/s41388-021-01801-w PMC8154592 33947960

[52] Zammataro L Lopez S Bellone S Pettinella F Bonazzoli E Perrone E. Whole-Exome Sequencing of Cervical Carcinomas Identifies Activating ERBB2 and PIK3CA Mutations as Targets for Combination Therapy. Proc Natl Acad Sci USA (2019) 116(45):22730–6. doi: 10.1073/pnas.1911385116 PMC6842590 31624127

[53] Liu N Mei L Fan X Tang C Ji X Hu X. Phosphodiesterase 5/Protein Kinase G Signal Governs Stemness of Prostate Cancer Stem Cells Through Hippo Pathway. Cancer Lett (2016) 378(1):38–50. doi: 10.1016/j.canlet.2016.05.010 27179930

